# Surgery of the Peritoneum, Etc.

**Published:** 1902-06-14

**Authors:** 


### SURGERY OF THE PERITONEUM, ETC.

**The Natural Method of Draining the Peritoneal Cavity** is thus summed up by Clarke[1]: (1) The absorbing function of the peritoneum is so great that it is capable of taking up 3 to 8 per cent. of the entire body weight in an hour. (2) Solid particles are quickly carried from the peritoneal cavity through the diaphragm into the mediastinal lymph vessels and glands, and thence into the blood circulation. (3) There is normally a force in the peritoneal cavity which carries fluids and foreign particles towards the diaphragm regardless of posture, although gravity may greatly favour or retard the current. (4) There is no possibility of limiting free infectious matter to any part of the peritoneal cavity by mechanical means. (5) In many cases, drainage as ordinarily employed is superfluous, or even dangerous, and the rational method is to remove all possible *débris* and infectious matter by thorough irrigation, and then leave one litre of salt solution (0·6 per cent.) in the abdominal cavity, and in order to promote and hasten natural drainage, supplement this by an enema of a litre of salt solution, given while the patient is well under anæsthesia. (6) Under this plan of treatment the patient is greatly stimulated, shock is minimised or averted, the urinary excretion is greatly increased, and thus toxic matters are more easily eliminated without irritation to the kidneys or bladder, peritoneal infection is quickly eliminated, thirst is alleviated or prevented, intestinal peristalsis is promoted, and consequently tympanites is of less frequent occurrence, and the early action of the intestine evacuates infectious matter thrown out into the canal by the blood-vessels of the villi. Welch[2] objects to drainage tubes after abdominal operations, because the tube keeps tissues asunder which might otherwise unite, and because the chance of bacterial invasion is greater in more ways than one. Besides increasing the chances of infection, drainage not infrequently gives rise to various post-operative complications. Of these Clarke[3] mentions: (1) obstruction of the bowel; (2) fæcal fistula; (3) vesical complications; (4) post-operative hernia. The methods for prevention or the removal of infective material without the employment of drainage may be summarised as follows: (1) a thoroughly aseptic technique; (2) the controlling of all hæmorrhage and oozing as far as possible; (3) careful manipulation, so that all unnecessary bruising of the tissues is avoided; (4) a perfect toilet of the peritoneal cavity; (5) the removal as far as possible of infectious foci; (6) the use of irrigations with salt solution; (7) if necessary the re-opening and thorough cleansing of the cavity; (8) proper after-care of the patient.

**Prevention of Stitch Abscess** is best accomplished, according to Maylard,[4] by sterilisation of the skin prior to operation by inunctions of oleate of mercury. The following is the plan he recommends:—(1) Cleanse the skin in the usual way by soap and water (turpentine and alcohol, or ether if necessary). (2) Anoint freely and widely with hydrated lanoline-oleate of mercury (20 per cent.) and rub in; besmear a piece of lint with the same and leave on until a second inunction is performed twelve hours later. Every case should be treated for at least 24 hours before operation—preferably 48 should be given—with at least two separate periods of "rubbing in" for about ten minutes on each occasion. 3. On the operating table the piece of lint is removed, and the superfluous ointment rubbed off with a piece of sterilised gauze. The part is now ready for operation.

**Pneumococcal Infection of the Peritoneum** is, says Barling[5] often associated with involvement of the pleura. It is important to differentiate between pneumococcal infections and other varieties, especially from those arising in the vermiform appendix, for the operative treatment is different. The points strongly in favour of a diagnosis of pneumococcal infection are: 1. The age of the patients, who are generally young children, as a rule of the female sex. 2. The onset may be associated with general body and limb pains. 3. Diarrhœa is generally present, instead of the constipation so common in appendicitis. 4. There is no particular localising pain as in appendical mischief. When the pus is evacuated it is odourless, and of a greenish colour, and there is a considerable tendency for it to find an escape at the umbilicus if it is left unrelieved. The treatment required is evacuation by incision in the middle line of the abdomen, careful irrigation and drainage.

**Thrombosis of the Mesenteric Vessels,** as a sequel to operation, is a very fatal affection. Maylard[6] thinks that the following symptoms when occurring within a few days after an operation may be taken as suggesting post-operative thrombosis of the mesenteric vessels, and more particularly so when the operation was not one involving the intestines: (1) the onset of intra-abdominal pain, gradual or acute, but more or less constant, and possibly of a colicky character; (2) neither tenderness or palpation of the abdomen, nor distension nor rigidity of the parietes in the earlier stages; (3) possibly diarrhœa with or without blood; (4) possibly vomiting, but not of the usually acute obstructive character; (5) rapidity of pulse; (6) undue and inexplicable restlessness and excitability; (7) any pre-existing symptoms of cardiac or vascular disease may be considered to attach additional weight to the significance of the other symptoms.

**Embolism of the Superior Mesenteric Artery** often gives rise to ileus. Borzeky[7] publishes an important case where acute intestinal obstruction occurred in a man who was the subject of mitral obstruction, and insufficiency not discovered till after death. The patient was seized with symptoms of obstruction with the passage of bloody stools in the early stage. At the operation a large amount of small intestine was found in an almost gangrenous condition, and an artificial anus was made as it was impossible to resect the gut. At the necropsy an embolism was found in the superior mesenteric artery, there was universal peritonitis, and nearly seven feet of small intestine were deep purple through hæmorrhage infarction. The author points out that in 14 out of 49 similar cases the patient passed for a time bloody stools; in the remainder obstruction was complete from the first; on that account Nothnagel defines two varieties of thrombosis and embolism of the mesenteric vessels.

**Gunshot Wounds of Abdomen.**—Da Costa[8] says that some of the most important principles in the treatment of these grave lesions are the following:—(1) Remove the patient at once to the nearest place where a clean operation may be undertaken. (2) Assure yourself positively that penetration has taken place. (3) Having demonstrated this fact, always open the abdomen and search for injuries, and make this search systematic. (4) Never wait for symptoms to tell you that profuse hæmorrhage or intestinal perforation has taken place, for by that time operation will usually be useless. (5) Cleanliness and thoroughness must never be sacrificed to speed. These two are absolute essentials of success, while speed is no longer an important factor since the introduction of normal saline solution Fenner[9] does not think that the high rate of mortality should discourage operation in gunshot and stab wounds of the abdomen. A mortality of 73·95 per cent. from gunshot wounds with injury of the hollow viscera means, in his opinion, that 26 per cent. of these patients had their lives saved by the operation; and a death rate of 33·33 per cent. from stab wounds, with the bowels cut, means that two-thirds were saved from certain death.

[1] Brit. Med. Jour., March 1, 1902; Epitome, No. 137; and Univ. of Penn. Med. Bull., Nov., 1901. [2] Amer. Jour. Med. Sci., Oct., 1901, p. 475. [3] Ibid., p. 476. [4] Ann. of Surg., Jan., 1902. Birm. Med. Rev., Dec., 1901, p. 350. [6] Brit. Med. Jour., Nov. 16, 01, p. 1454. [7] Brit. Med. Jour., Jan. 25, 1902. Epitome, No. 50. Amer. Jour. Med. Sci, Dec., 1901, p. 717. [9] Med. Rec., Nov. 30, 01, p. 871.

